# Impact of different breathing conditions on the dose to surrounding normal structures in tangential field breast radiotherapy

**DOI:** 10.4103/0971-6203.31146

**Published:** 2007

**Authors:** Ramachandran Prabhakar, Ganesh Tharmar, Pramod K. Julka, Goura K. Rath, Rakesh C. Joshi, Anil K. Bansal, R. K. Bisht, N. Gopishankar, G. S. Pant, S Thulkar

**Affiliations:** Department of Radiotherapy, Institute Rotary Cancer Hospital, All India Institute of Medical Sciences, New Delhi, India; * Department of Nuclear Medicine, All India Insitute of Medical Sciences, New Delhi, India; ** Department of Radiology, All India Institute of Medical Sciences, New Delhi, India

**Keywords:** Breast cancer, cardiac dose, organ motion

## Abstract

Cardiac toxicity is an important concern in tangential field breast radiotherapy. In this study, the impact of three different breathing conditions on the dose to surrounding normal structures such as heart, ipsilateral lung, liver and contralateral breast has been assessed. Thirteen patients with early breast cancer who underwent conservative surgery (nine left-sided and four right-sided breast cancer patients) were selected in this study. Spiral CT scans were performed for all the three breathing conditions, viz., deep inspiration breath-hold (DIBH), normal breathing phase (NB) and deep expiration breath-hold (DEBH). Conventional tangential fields were placed on the 3D-CT dataset, and the parameters such as V30 (volume covered by dose >30 Gy) for heart, V20 (volume covered by dose >20 Gy) for ipsilateral lung and V_50_ (volume receiving >50% of the prescription dose) for heart and liver were studied. The average reduction in cardiac dose due to DIBH was 64% (range: 26.5-100%) and 74% (range: 37-100%) as compared to NB and DEBH respectively. For right breast cancer, DIBH resulted in excellent liver sparing. Our results indicate that in patients with breast cancer, delivering radiation in deep inspiration breath-hold condition can considerably reduce the dose to the surrounding normal structures, particularly heart and liver.

Treatment of early breast cancer by radiotherapy after conservative surgery improves local control. However, improvement in treatment outcome must always be balanced with the potential risk of long-term complications such as late cardiac mortality and radiation-induced pneumonitis. The challenging parameters which interfere in achieving the treatment outcome and complications are organ motion and setup-errors. Movement of the target volume has increasingly been considered as an important factor in treatment planning. The internal organ contours are prone to change during respiration. As the rib cage and lung expands during inspiration, the distance between the heart and chest wall increases. There are limited quantitative data in the literature on organ movement. The use of spiral computerized tomography (CT) requires an additional consideration in the delineation of the planning target volume (PTV). These images are acquired in a single breath-hold, unlike CT images obtained with standard scanner.

A very recent cohort study by Darby *et al.* on long-term mortality from heart disease and lung cancer after radiotherapy for early breast cancer in about 300,000 women in the US shows that radiotherapy can increase mortality from heart disease and lung cancer.[1] The mean cardiac dose from irradiation of a left-sided breast cancer can be two or three times that for a right-sided breast cancer, and the mean ipsilateral lung dose can also be two or three times the mean contralateral lung dose. Similar studies of radiation toxicity in the treatment of breast cancer have shown that the effects on normal tissues can constitute a significant clinical problem, particularly increased cardiac mortality, and this may offset any potential survival benefit of treatment.[2–4] The patients with lower risk of dying from breast cancer are at the highest risk of cardiac mortality due to their longer relapse-free survival.[5] Retrospective cohort studies show that the patients who are most at risk of cardiac mortality are those requiring radiotherapy to left breast.[6,7]

In this study, we have investigated the impact of deep inspiration breath-hold (DIBH), deep expiration breath-hold (DEBH) and normal breathing (NB) on the dose to surrounding structures like heart, lung, liver and contralateral breast (CLB). Also the planned isocenter shift between the normal breathing and deep inspiration breath-hold has been assessed and correlated with cardiac dose reduction in tangential field breast radiotherapy.

## Materials and Methods

Thirteen patients with early breast cancer who underwent conservative surgery were selected for this study. For studying the cardiac and the liver dose, nine left-sided and four right-sided breast cancer patients were inducted in this study. Prior to imaging, the patients were trained to hold their breath in deep inspiration and deep expiration. Thin copper wires were placed along the medial and lateral field borders at the time of image acquisition, serving as guiding tools for the field placement. The patients were positioned with their arms abducted on the treatment side to 90°. The area of CT scanning included the superior (cranial) and inferior (caudal) border of the field marked by the radiation oncologist with an addition of 3 cm margin. Spiral CT scans were performed in Siemens Volume Zoom CT with 3-mm slice thicknesses for all the three breathing conditions, viz., DIBH, NB and DEBH. In our study, in normal breathing phase, the CT was acquired as performed routinely. The average time for which the patients were asked to hold their breath was 18 s. The CT image data sets were then transferred to the eclipse treatment planning system through DICOM network. Structures such as body (external contour), PTV, ipsilateral lung (IL), heart, CLB and liver were delineated on NB, DIBH and DEBH reconstructed 3D-CT datasets. For simplicity in comparing the three different breathing conditions, the delineation of PTV was based on the medial and lateral marker wires placed on the skin during image acquisition. The PTV was delineated from a straight line drawn between the lateral and medial marker and growing it to the anterior and lateral skin surface. The chest wall was subtracted from the grown PTV, and similarly the body was also subtracted with a 3-mm margin between the skin surface and PTV. Conventional tangential fields were placed on the 3D-CT dataset using isocentric technique with matching posterior field borders. The isocenter was placed at the center of mass of the PTV with the help of the tool available in the planning system. The isocenter coordinates for all the three breathing conditions were noted down with respect to the DICOM image origin. The 3D shift between the isocenter of two breathing conditions (NB, DIBH) was computed with the distance formula, and it is called the planning isocenter shift. The plan was normalized to the isocenter (prescription dose: 50 Gy/25#). For each patient, simple tangential field plans were created for the three different CT data sets, and DVH analyses were performed for the following structures: PTV, heart, ipsilateral lung, liver and CLB. Parameters such as V30 for heart, V20 for ipsilateral lung and V_50_ for heart and liver were studied.

## Results

**Figure 1:**
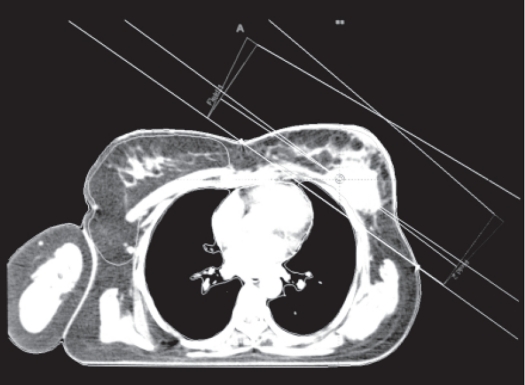
Tangential field planning for NB

**Figure 2:**
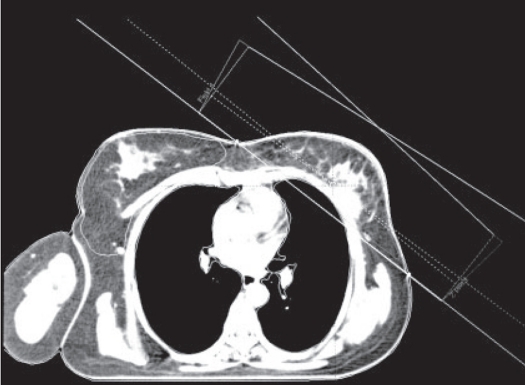
Tangential field planning for DIBH

**Figure 3:**
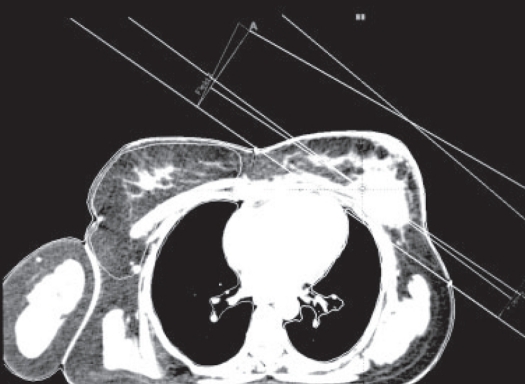
Tangential field planning for DEBH

**Figure 4:**
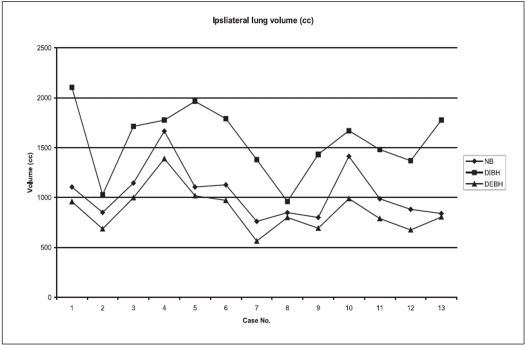
Ipsilateral lung volume

**Figure 5:**
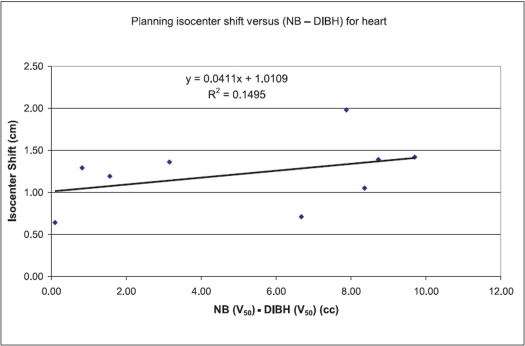
Planning isocenter shift versus V_50_ (NB - DIBH) for heart

Figures 1–3 show the tangential field technique used for planning NB, DIBH and DEBH respectively. Table 1 shows the cardiac (left-sided breast) dose volume parameters (V_50_ - volume receiving >50% of the prescription dose) for NB, DIBH and DEBH. The mean cardiac volume covered by V30 for NB, DIBH and DEBH was 13.08 cc, 6.78 cc and 19.89 cc respectively. The results indicate a considerable reduction in the cardiac dose due to DIBH as compared to NB and DEBH. The average reduction in cardiac dose due to DIBH was 64% (range: 26.5-100%) and 74% (range: 37-100%) as compared to NB and DEBH respectively. Figure 4 shows the ipsilateral lung volume in all the three breathing conditions (NB, DIBH and DEBH). There is a considerable increase in the lung volume from DEBH to NB and also from NB to DIBH. The ipsilateral lung volume ranged between 760.7 cc and 1,666.7 cc (Median: 987.2 cc, Mean: 1,041.58 cc ± 263.48 cc), 962.1 cc and 2,104.6 cc (Median: 1,670 cc, Mean: 1,573.32 cc ± 337.70 cc), 564.7 cc and 1,388.3 cc (Median: 804.74 cc, Mean: 872.56 cc ± 214.29 cc) for NB, DIBH and DEBH respectively. Similarly, the mean lung volume covered by V20 for NB, DIBH and DEBH was 13.9, 12.2 and 15.2% respectively. Table 2 shows the liver (right-sided breast) dose volume parameters (V_50_ - volume receiving >50% of the prescription dose) for NB, DIBH and DEBH. The average reduction in liver dose due to DIBH was 50.22% (median: 54.34%) and 70.63% (median: 67.66%) as compared to NB and DEBH. Dose to contralateral lung was not affected by different breathing conditions. For right breast cancer, DIBH resulted in excellent liver sparing. Figure 5 shows the relationship between the planning isocenter shift and difference between values of V_50_ observed for heart during NB and DIBH. The isocenter shift increased with the increase in V_50_ difference between NB and DIBH (r = 0.39). The maximum 3D isocentric shift between NB and DIBH was 2 cm with a median value of 1 cm.

**Table 1:** Dose volume parameters for heart

*Cardiac parameters*	*NB-V_50_ (cc)*	*DIBH-V_50_ (cc)*	*DEBH-V_50_ (cc)*
Range	0.1 - 25.17	0 - 18.5	1.56 - 30.61
Mean ± stdev.	9.86 ± 8.26	4.65 ± 5.86	14.38 ± 9.94
Median	10.05	3.15	14.84

NB - Normal breathing, DIBH - Deep inspiration breath-hold, DEBH - Deep expiration breath-hold

**Table 2:** Dose volume parameters for liver

*Liver parameters*	*NB-V_50_ (cc)*	*DIBH-V_50_ (cc)*	*DEBH-V_50_ (cc)*
Range	4.03 - 31.48	0 - 29.78	5.69 - 73.24
Mean ± stdev.	14.57 ± 11.88	9.34 ± 13.8	28.8 ± 30.5
Median	11.39	3.79	18.14

NB - Normal breathing, DIBH - Deep inspiration breath-hold, DEBH - Deep expiration breath-hold

## Discussion

Lung and cardiac toxicity are two potential risk elements of breast radiotherapy. Among the two, cardiac damage is recognized to be a potentially serious side effect of breast cancer radiotherapy. The level of cardiac toxicity is dependent on the technique used for treatment. Respiration affects the position of the breast, lungs and heart. Standard CT acquisition captures a static image in a moment of time. At that moment, it is unknown whether the image is acquired during inspiration or expiration. Gating can be achieved either using breath-hold techniques or normal respiration. This can minimize the lung motion and to some degree cardiac motion.

Gagliardi *et al.* investigated the relationship between risk of cardiac mortality data for patients in RT *vs.* no RT studies, and they found that individual patients might have an absolute risk of cardiac death as high as 9%, depending on the volume of heart treated.[3,8] There are studies showing increase in late cardiac mortality for left-sided breast when compared to right-sided breast.[7,9] Hurkmans *et al.* applied the same model to tangential irradiation and found that increasing maximum heart depth predicted increased risk of excess cardiac mortality.[10] Modern breast conservation therapy should be able to optimize the therapeutic ratio, preserving high tumor control rates with minimal late cardiac toxicity. A study by Balter *et al.* showed that free-breathing CT may improperly estimate the position and volume of the critical structures and may mislead evaluation of plans.[11] It has been demonstrated that a reduction in cardiac volume irradiated can be achieved by treating patients during deep inspiration. Several authors have stated that deep inspiration breath-hold technique favors the sparing of heart dose when compared to deep-expiration and normal breathing conditions.[12–15] There are several techniques for achieving the deep inspiration breath-hold conditions; of these, some of the common approaches are voluntary breath-hold technique,[13,15,16] controlled breath-hold technique (active breathing coordinator),[12,16,17] breathing-adapted (gated) radiotherapy.[15] Each of the above techniques has its own advantages and disadvantages. Introduction of active breathing coordinator (ABC) reduced the V30 for heart in DIBH conditions.[12] Pederson *et al.* found that for right-sided tumors, the median ipsilateral relative lung volume receiving >50% of the prescribed CTV dose was 39, 49 and 32% for free-breathing (FB), expiration breath-hold (EBH) and DIBH respectively; and for left-sided tumors, the corresponding percentages were 37, 46 and 31% using breathing-adapted radiotherapy.[15] The median heart volume receiving >50% of the prescription dose was reduced from 8% for FB to 1% for DIBH. Our results indicate similar findings in carcinoma breast patients - that delivering radiation in inspiration breath-hold condition can considerably reduce the dose to the surrounding normal structures, particularly, heart and liver.

The median cardiac volume covered by the dose >25 Gy was 10.05 cc, 3.15 cc and 14.84 cc for NB, DIBH and DEBH respectively, which clearly indicates that the cardiac dose was significantly reduced in DIBH. Similarly, for ipsilateral lung, DIBH resulted in reduced dose. For right breast cancer, DIBH resulted in excellent liver sparing. On correlating the isocenter shift with the spared cardiac volume due to DIBH (V_50_ for NB - V_50_ for DIBH), a positive correlation (r = 0.39) was observed. The positive correlation indicates that the cardiac dose spared by DIBH has a relation with planning isocenter shift and it increases with increase in isocenter shift. The correlation of the isocenter shift with the liver volume spared by DIBH when compared to NB (NB-DIBH) was shown to be positive (r = 0.293). Though the number of right-sided breast patients selected for this study is less, our results show a positive correlation with reduction in the dose to the liver. An important concern in treating the breast with tangential field is the movement of target volume with respiration. In DIBH, as patients take a deep breath the lung expands and displaces the heart away from the left chest wall and also more inferiorly. This results in reduction in the cardiac dose. As the rib cage expands, the PTV also gets displaced away from its position. Since, the isocenter is placed at the center of mass of the target volume, the isocenter also gets displaced. Though the machine isocenter is fixed, but with respect to the patient, the planned isocenter gets shifted because of respiration and it affects the dose homogeneity and the dose to surrounding structures. Hence, treating the patient in DIBH avoids the unexpected respiratory motion during treatment and also reduces the normal tissue toxicity.[18,19]

This is a preliminary study using voluntary breath-hold technique in assessing the dose to surrounding normal structures; it also shows that with proper training in controlling the breathing during treatment, it may reduce long-term cardiac toxicity in DIBH. If the patient can hold the breath for 21 s, 140 MU for typical medial or lateral tangential field with 400 MU/min can be delivered safely using the DIBH technique.

Modern breast conservation therapy should have the capability to optimize the therapeutic ratio, preserving high tumor control rates with minimal late cardiac toxicity. In the absence of large prospective clinical trials and with insufficiently long follow-up, it is unclear what level of cardiac irradiation is unacceptable. Therefore, our current aim should be to reduce any high-dose cardiac irradiation, with good target coverage. Image-guided radiation therapy equipped with gating or active breathing coordinator helps in combating the problems due to organ motion, especially in breast radiotherapy.

## Conclusion

Organ motion is one of the serious concerns in radiotherapy, and with the induction of newer treatment approaches like image-guided radiotherapy, the importance of organ motion in treatment planning has been highlighted. The positive correlation of the planning isocenter shift with NB and DIBH shows that DIBH displaces the heart away from the planning isocenter; and the larger the shift in isocenter, the greater is the reduction in the dose to the heart. The three different breathing conditions simulated for nine patients showed that the cardiac volume could be considerably reduced by DIBH for left-sided breast treatment. Similarly, the study also showed that liver could be spared to a larger extent with DIBH for right-sided breast cancer. Our results indicate that in patients with breast cancer, delivering radiation in inspiration breath-hold condition can considerably reduce the dose to the surrounding normal structures, particularly the heart and liver.
